# Overexpression of Grapevine *VyTRXy* Improves Drought Tolerance by Maintaining Photosynthesis and Enhancing the Antioxidant and Osmolyte Capacity of Plants

**DOI:** 10.3390/ijms242216388

**Published:** 2023-11-16

**Authors:** Jiang Xiang, Min Li, Yiyi Li, Yi Liu, Lingzhu Wei, Ting Zheng, Jiang Wu, Yihe Yu, Jianhui Cheng

**Affiliations:** 1 Institute of Horticulture, Zhejiang Academy of Agricultural Sciences, Hangzhou 310021, China; xiangjiang@zaas.ac.cn (J.X.); weilingzhu@zaas.ac.cn (L.W.); zhengting12309@163.com (T.Z.); wujiang@zaas.ac.cn (J.W.); 2 Henan Engineering Technology Research Center of Quality Regulation and Controlling of Horticultural Plants, College of Horticultural and Plant Protection, Henan University of Science and Technology, Luoyang 471023, China; limin03301@163.com (M.L.); liyiyi0153@163.com (Y.L.); liuyi_0801@163.com (Y.L.); yuyihe@haust.edu.cn (Y.Y.)

**Keywords:** grapevine, *VyTRXy*, drought, photosynthesis

## Abstract

Drought stress profoundly affects plant growth and development, posing a significant challenge that is extensively researched in the field. Thioredoxins (TRXs), small proteins central to redox processes, are crucial to managing both abiotic and biotic stresses. In this research, the *VyTRXy* gene, cloned from wild Yanshan grapes, was validated as a functional TRX through enzyme activity assays. VyTRXy was found to bolster photosynthesis, augment levels of osmotic regulators, stimulate antioxidant enzyme activities, and strengthen drought resilience in transgenic plants. These enhancements were evidenced by higher survival rates, optimized photosynthetic metrics, increased proline levels, augmented chlorophyll concentration, reduced electrolyte leakage, and decreased malondialdehyde and hydrogen peroxide (H_2_O_2_) levels. Furthermore, there was a surge in the activities of enzymes such as catalase, ascorbate peroxidase, glutathione peroxidase, dehydroascorbate reductase, and glutathione reductase, along with an increased expression of TRX peroxidase. Notably, under drought stress, there was a marked elevation in the expression of stress-responsive genes, including the adversity stress-inducible expression gene (*NtRD29A*) and DRE-binding protein (NtDREB), in transgenic tobacco. This investigation is pivotal in the quest for drought-resistant grapevine varieties and provides significant insights into the molecular functionality of *VyTRXy* in enhancing grapevine drought tolerance.

## 1. Introduction

Thioredoxins (TRXs) are small proteins that participate in redox processes. They have conserved redox activity and are pivotal in the redox regulation of organisms by reducing disulfide bridges in target proteins. Essential for abiotic and biotic stress tolerance [1], TRXs function in a broad spectrum of metabolic processes as oxidoreductases and regulate numerous chloroplast enzymes and photosynthesis. Research indicates that TRXs may affect plant drought stress tolerance [2]. The TRX protein family includes six types: h, o, f, m, x, and y, with f, m, x, and y localized within the chloroplasts [3]. TRXx and TRXy have been recognized as effective reducers of various peroxide-reducing proteins in the antioxidant system [4]. In *Arabidopsis thaliana*, TRXy comprises two main components: TRXy1 and TRXy2. TRXy1 acts as the reducing agent for TRX, while TRXy2 is associated with photosynthesis [5].

Drought is a major abiotic stressor and a significant limitation on global crop yields [6]. Plant response to drought stress is a complex process that upsets the balance of reactive oxygen species (ROS), leading to hindered growth and even programmed cell death [7]. Drought stress prompts leaf senescence, adversely affecting plant growth by curtailing photosynthesis [8]. In grapes, drought significantly affects berry and wine quality and grapevine yield [9,10]. Drought stress reduces plant photosynthesis, affecting growth and development, causing leaf desiccation and fruit abscission [11]. Chinese-domesticated watermelon shows leaf wilting and yellowing under drought stress due to significant decreases in photosynthetic photosystem II efficiency, initial Rubisco activity, and chlorophyll concentration [12]. Plants produce considerable amounts of ROS under adverse conditions like drought, high temperatures, and intense light, with the levels of ROS regulated by various factors, the redox system being a principal one.

Grapes, globally valued as a fruit crop, have extensive applications across various food and beverage industries. Their cultivation yields significant economic advantages while also enriching the social and ecological facets of the industry. However, grape growth and development are profoundly affected by abiotic stresses, especially drought. Drought stress can cause physical damage to roots, leaves, and other grape organs, reducing yield and potentially leading to plant death [13]. To bolster drought resistance in cultivated grape varieties, it is vital to explore and utilize the drought resistance genes in the Chinese wild grape, a germplasm resource known for its exceptional drought tolerance.

Chloroplasts, the primary sites for photosynthesis in plants, act as central hubs for sensing signals initiated by external biotic and abiotic stresses, helping plants withstand unfavorable environmental conditions [14]. Studies highlight chloroplasts’ vulnerability to drought stress, with related proteins contributing to the plant’s stress response [15]. Abscisic acid (ABA) serves as a key signal for plants under drought stress, instigating various physiological processes like stomatal closure and significantly influencing plant growth and development [16]. In rice, the chloroplast-localized Os3BGlu6 protein helps regulate cellular ABA levels, affecting drought tolerance and photosynthesis [17]. The overexpression of the *GhTRX134* gene in cotton bolsters resistance to abiotic stresses and enhances drought and salt tolerance in transgenic *Arabidopsis* plants [18]. TRX, under drought stress, engages in redox signaling and the scavenging of ROS, promoting detoxification responses and maintaining redox homeostasis [19].

Our study successfully isolated the *VyTRXy* gene from *Vitis yeshanensis* grapes, and enzyme activity assays confirmed its function as a TRX. Tissue-specific analyses showed notable *VyTRXy* expression in the roots. We also discovered that drought stress significantly stimulates *VyTRXy* expression, resulting in the accumulation of stress-related compounds and the activation of stress-related genes in transgenic tobacco. Our research lays the theoretical groundwork for investigating the drought resistance mechanisms in grapevine and breeding more drought-resistant grapevine varieties, and proposes new solution directions for improving photosynthesis and enhancing drought stress tolerance in grapevine, and providing genetic resources for grapevine germplasm innovation.

## 2. Results

### 2.1. Expression Patterns of VyTRXy under Stress Treatments

**Figure 1:**
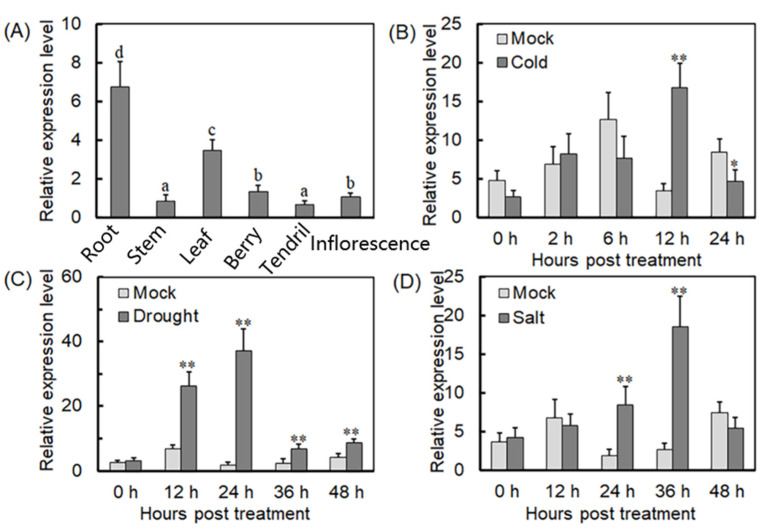
Expression analysis of *VyTRXy* in grapes. (**A**) Expression of *VyTRXy* in different tissues of grapes. (**B**–**D**) Expression of *VyTRXy* in grapes after cold (**B**), drought (**C**), and salt (**D**) treatments. The asterisk indicates significant differences between different treatments (* *p* < 0.05, ** *p* < 0.01, Student’s *t*-test). The above experiment was repeated three times, and the results were similar. a–d indicate whether there is a significant difference between two comparisons, with the same letter indicating no difference and different letters indicating a difference. They are labeled in order, with a greater difference in order indicating a more significant difference between the two.

*VyTRXy* expression was analyzed using quantitative reverse transcription–polymerase chain reaction (qRT-PCR) across different grapevine organs and under various stress conditions. It was detected in all grapevine organs, with the highest expression in roots, followed by leaves (Figure 1A). Relative to the control, *VyTRXy* expression was significantly upregulated at 12 h (h) during cold stress treatment (Figure 1B). *VyTRXy* expression was significantly elevated under drought stress (Figure 1C). *VyTRXy* expression was significantly increased at 24 and 36 h in plants treated with high salt (Figure 1D). These outcomes suggest that *VyTRXy* expression is inducible by abiotic stress, showing particularly high sensitivity to drought stress.

### 2.2. Enzymatic Activity and Subcellular Localization of VyTRXy

**Figure 2:**
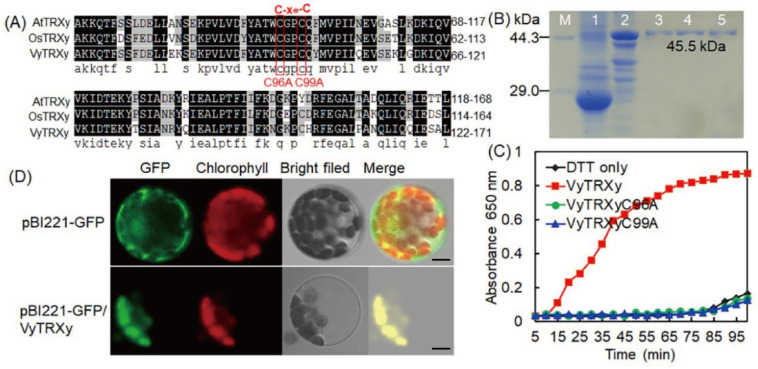
Biological functional analysis of VyTRXy. (**A**) Multiple sequence alignment analysis of *VyTRXy* with *AtTRXy* and *OsTRXy*. The Cys residue (Cys96 and Cys99) sites are marked in red, and the black and gray backgrounds indicate the degree of similarity of the sequence comparison. * indicates another amino acid. (**B**) Determination of the relative molecular mass of proteins. Lane M: protein labeling; 1: uninduced empty vector; 2: induced vector containing *VyTRXy*; lanes 3, 4 and 5: purification of lane 2. (**C**) Determination of VyTRXy enzyme activity. (**D**) Distribution of pBI221-GFP/VyTRXy and control pB1221-GFP under laser confocal microscopy; GFP—green fluorescence signal; Chlorophyll—red fluorescence signal; Bright field—light field map; Merge—merged map. Bars, 200 μm.

A multiplex sequence comparison showed a highly conserved structure among VyTRXy, AtTRXy, and OsTRXy (Figure 2A). The VyTRXy protein had a molecular weight of 16.5 kDa, aligning with gel electrophoresis results (Figure 2B). Enzyme activity assays indicated that the single mutations C96A and C99A led to a complete loss of enzyme activity (Figure 2C). This evidence confirms that VyTRXy encodes a functional TRX and underscores the critical role of Cys residues (Cys96 and Cys99) in its catalytic activity. Laser confocal microscopy showed intense green fluorescence in chloroplasts of plants transfected with pBI221-GFP/VyTRXy, in contrast to the universal fluorescence in *Arabidopsis* protoplasts containing the pB1221-GFP control vector.

### 2.3. Overexpression of VyTRXy Enhanced Drought Resistance in Transgenic Tobacco

**Figure 3:**
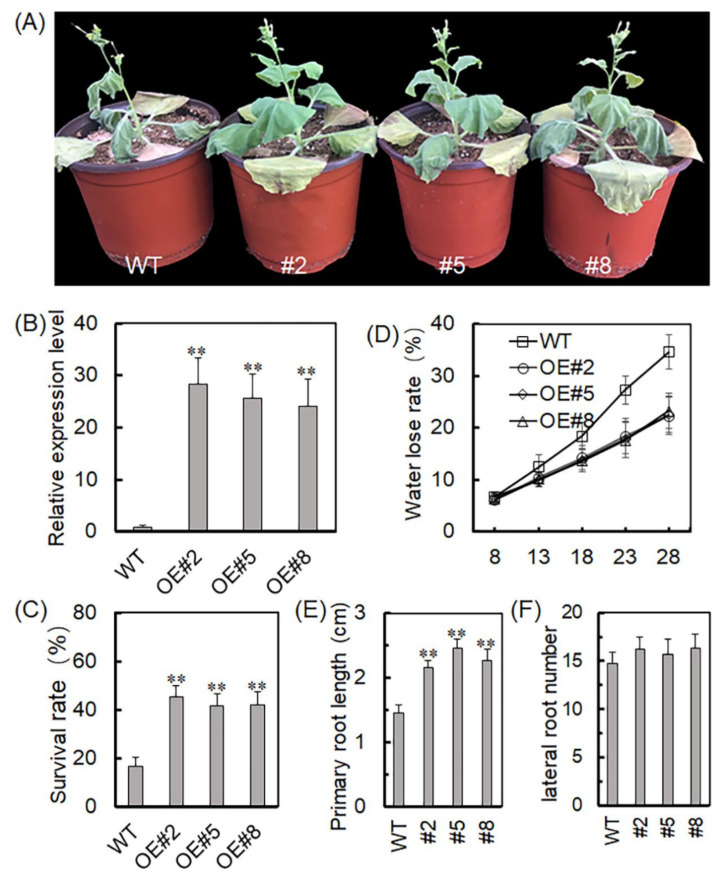
Reduced sensitivity to drought in *VyTRXy* transgenic plants overexpressing *VyTRXy*. (**A**) Phenotypes of transgenic lines OE#2, OE#5, and OE#8 and WT plants after drought treatment. (**B**) Transcript levels of *VyTRXy* in transgenic strains and WT plants. (**C**,**D**) Determination of survival rate and water loss in transgenic plants and control plants after drought treatment. (**E**,**F**) Determination of primary root lengths and lateral root number in transgenic and control plants after drought treatment. Differences between treatments are indicated by an asterisk indicating the degree of difference (** *p* < 0.01, Student’s *t*-test). All of the above experiments were repeated three times, and similar results were obtained.

To further investigate *VyTRXy*’s role in plant growth and development, an overexpression vector was utilized (Tabe S1), and three transgenic tobacco lines were developed through ectopic expression (Figure 3A). The qRT-PCR assay confirmed positive expression in OE#2, OE#5, and OE#8 lines of the transgenic plants(Figure 3B). *VyTRXy* expression was further analyzed in the transgenic plants under drought stress, revealing significantly higher levels than those in the wild-type (WT) plants. The water loss rate in WT plant leaves was significantly higher than that in the transgenic lines (Figure 3D). Phenotypic analysis showed the transgenic lines had longer primary roots than the WT following drought stress treatment, though there was no significant difference in the number of lateral roots (Figure 3E,F). Moreover, the WT plants displayed greater drought sensitivity and lower survival rates than the transgenic lines (Figure 3C).

**Figure 4:**
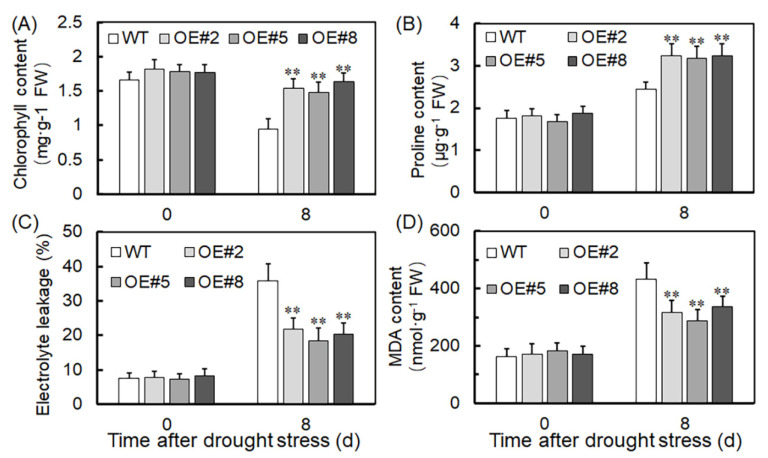
Determination of drought resistance of *VyTRXy* in tobacco. (**A**) Chlorophyll content. (**B**) Proline content. (**C**) Electrolyte leakage. (**D**) Malondialdehyde content in leaves. Differences between treatments are indicated by an asterisk indicating the degree of difference (** *p* < 0.01, Student’s *t*-test). All of the above experiments were repeated three times, and similar results were obtained.

To assess the impact of physiological changes under drought stress conditions, plant stress resistance-related indicators were evaluated. After 8 days of drought treatment, the transgenic plants showed significantly higher levels of chlorophyll and proline accumulation than the WT (Figure 4A,B). Conversely, the transgenic plants had significantly lower levels of electrolyte leakage and malondialdehyde (MDA) content than the WT (Figure 4C,D). These findings suggest that *VyTRXy* overexpression enhances the drought stress resistance in transgenic plants.

### 2.4. Overexpression of VyTRXy Promoted Photosynthesis in Transgenic Tobacco

**Figure 5:**
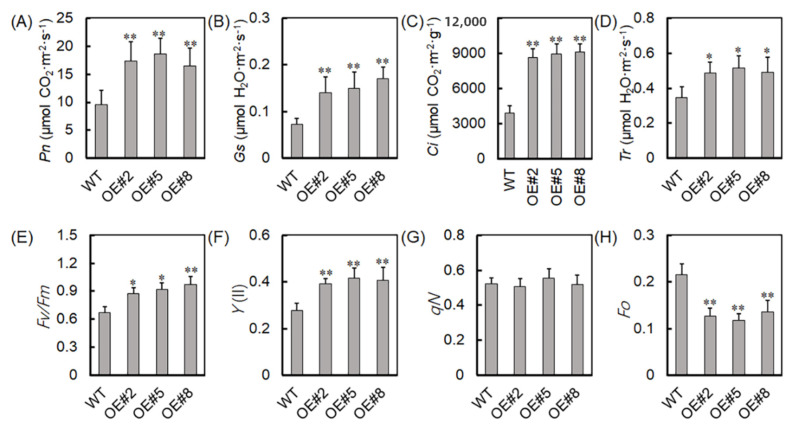
*VyTRXy* enhances drought tolerance by increasing photosynthesis in *transgenic tobacco*. (**A**) Pn—net photosynthesis. (**B**) Gs—stomatal conductance. (**C**) Ci—intercellular CO_2_ concentration. (**D**) Tr—transpiration rate. (**E**) Fv/Fm—the maximum photochemical efficiency. (**F**) Y(II). (**G**) qN. (**H**) Fo—fixed fluorescence. The asterisk indicates significant differences between different treatments (* *p* < 0.05, ** *p* < 0.01, Student’s *t*-test). The above experiment was repeated three times, and the results were similar.

The effect of *VyTRXy* on plant photosynthesis under drought stress was also examined by measuring photosynthesis-related parameters in transgenic tobacco using a photosynthesis analyzer. The transgenic tobacco plants OE#2, OE#5, and OE#8 demonstrated significant increases in net photosynthesis (Pn), stomatal conductance (Gs), intercellular CO_2_ concentration (Ci), and transpiration rate (Tr) compared with the WT plants, indicating that *VyTRXy* overexpression enhances photosynthesis in plants (Figure 5A–D).

For a deeper understanding of *VyTRXy*’s role in enhancing plant photosynthesis, the levels of initial fluorescence (F0), non-photochemical quenching (qN), maximum photochemical efficiency (Fv/Fm), and the quantum yield of PSII (Y(II)) were measured. The transgenic tobacco plants showed a significantly lower level of F0 compared with the WT (Figure 5H). By contrast, both Fv/Fm and Y(II) were significantly higher in the transgenic plants than in the WT plants (Figure 5E,F), while qN showed no significant differences (Figure 5G). These results suggest that *VyTRXy* may improve photosynthesis in plants by reducing F0 levels or enhancing the Fv/Fm and Y(II) pathways.

### 2.5. Overexpression of VyTRXy Regulates the Expression of Stress-Related Genes under Drought Treatment

**Figure 6:**
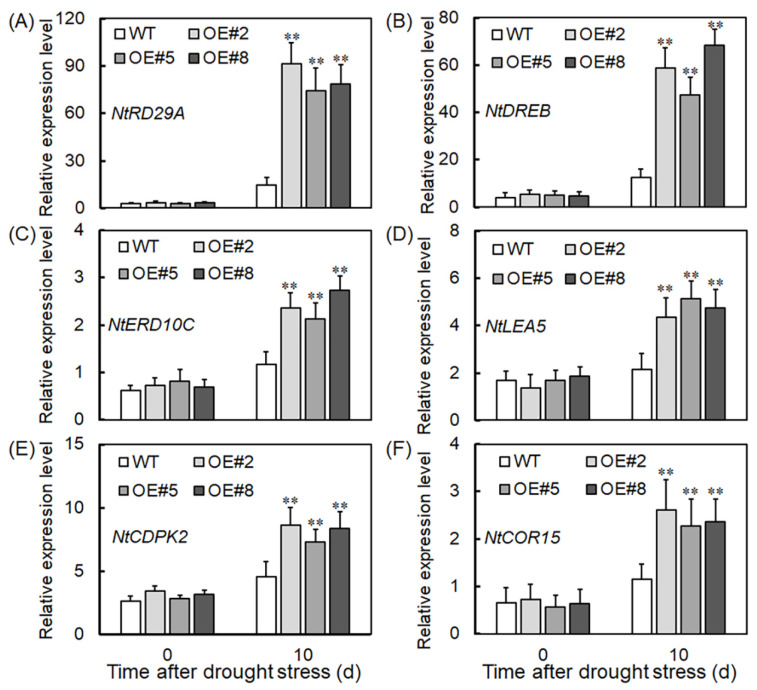
Relative expression of drought stress-related genes (*NtRD29A*, *NtDREB*, *NtERD10C*, *NtLEA5*, *NtCDPK2*, and *NtCOR15*) in transgenic plants and WT after drought treatment. (**A**) *NtRD29A*, (**B**) *NTDREB*, (**C**) *NtER10C*, (**D**) *NtLEA5*, (**E**) *NtCDPK2*, and (**F**) *NtCOR15*. Significant differences were indicated using the Student’s *t*-test (** *p* < 0.01).

To clarify *VyTRXy*’s role in plants’ response to drought stress, we analyzed the transcript levels of stress-related genes, including *NtRD29A*, *NtDREB*, *NtERD10C*, *NtLEA5*, *NtCDPK2*, and *NtCOR15*. qRT-PCR results revealed that all drought treatments elevated the expression of these genes, with transgenic lines showing significantly higher levels than the control group (Figure 6A–F). Notably, *NtRD29A* experienced the most pronounced increase in expression under drought conditions (Figure 6A), closely followed by *NtDREB* (Figure 6B). These results suggest that *VyTRXy* can elevate the expression of drought-related genes under stress, thereby bolstering plant drought resistance. Moreover, *VyTRXy* overexpression seems to primarily enhance drought resistance by elevating *NtRD29A* and *NtDREB* expression.

### 2.6. Overexpression of VyTRXy Increases Antioxidant Enzyme Activities and Decreases the H_2_O_2_ Content in Transgenic Tobacco under Drought Treatment

**Figure 7:**
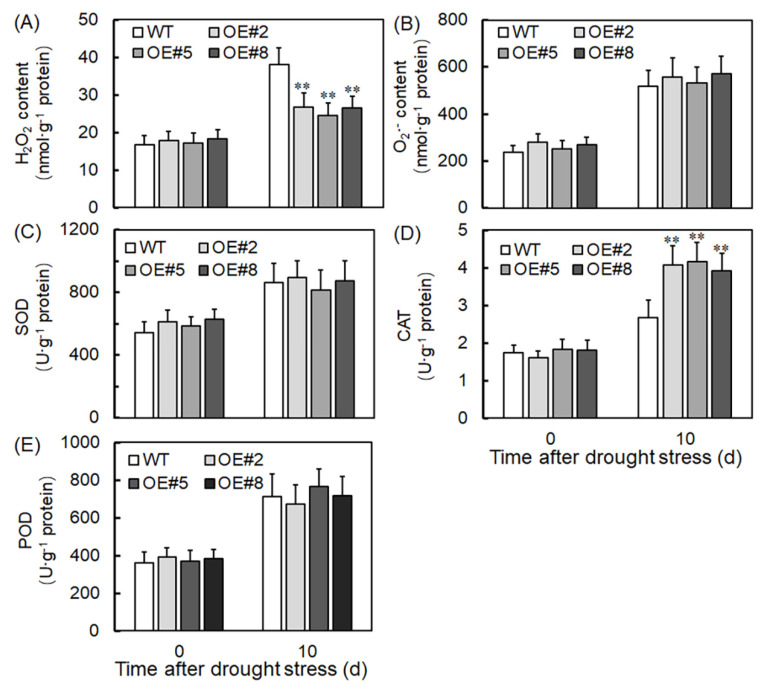
Oxidase activity and H_2_O_2_ content of transgenic tobacco plants under drought treatment. (**A**) H_2_O_2_. (**B**) O_2_^−^. (**C**) SOD. (**D**) CAT. (**E**) POD. Significant differences were indicated using the Student’s *t*-test (** *p* < 0.01).

To explore *VyTRXy* overexpression’s impact on drought resistance in grapes, we measured the levels of H_2_O_2_ and O_2_^−^ in transgenic plants subjected to drought stress. Transgenic plants displayed a significantly lower H_2_O_2_ content than the control group plants (Figure 7A), while the O_2_^−^ content was comparable to that of the WT plants (Figure 7B). These observations suggest that *VyTRXy* overexpression helps lower H_2_O_2_ content in plants. We also assessed the activities of crucial antioxidant enzymes, including superoxide dismutase (SOD), catalase (CAT), and peroxidase (POD), before and after drought exposure. While POD and SOD levels in transgenic plants remained relatively stable after drought exposure compared with controls (Figure 7C,E), CAT content significantly increased. This evidence implies that *VyTRXy* overexpression might boost CAT activity, thereby engaging the ROS pathway and indirectly diminishing hydrogen peroxide generation (Figure 7D). Notably, the increase in hydrogen peroxide levels in transgenic plants was significantly lower than that in WT plants, indicating that *VyTRXy* overexpression might curb hydrogen peroxide generation under drought stress, thus enhancing plants’ drought resistance.

**Figure 8:**
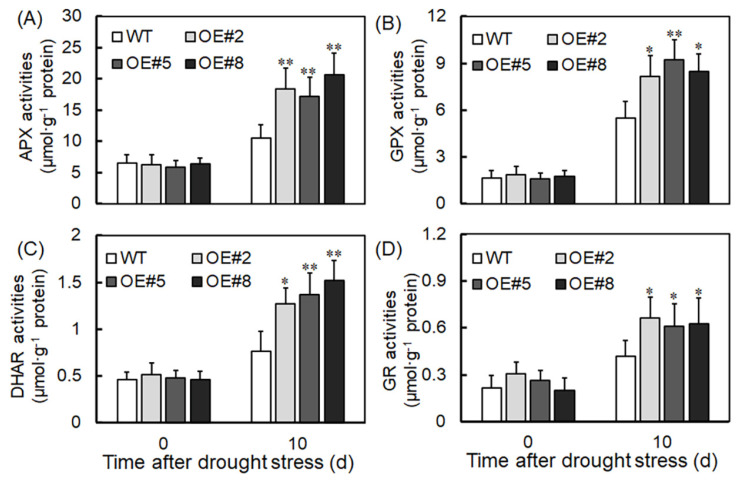
Determination of oxidoreductase activity in transgenic tobacco plants under drought treatment. (**A**) APX—ascorbate peroxidase. (**B**) GPX—glutathione peroxidase. (**C**) DHAR—dehydroascorbate reductase. (**D**) GR—glutathione reductase. Significant differences were indicated using the Student’s *t*-test (* *p* < 0.05, ** *p* < 0.01).

**Figure 9:**
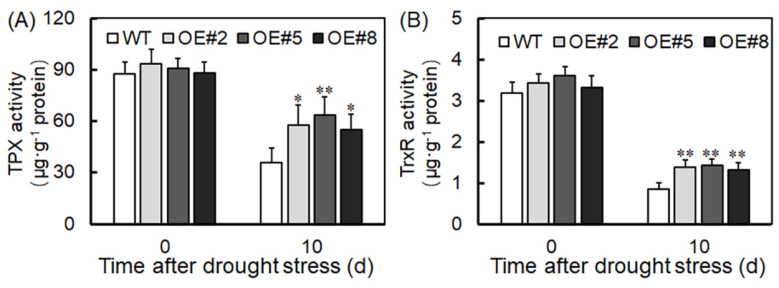
Relative expression of TRX and TrxR in transgenic plants after 10 days of drought treatment. (**A**) Relative expression levels of TRX in transgenic plants. (**B**) Relative expression levels of TrxR in transgenic plants. Significant differences were indicated using the Student’s *t*−test (* *p* < 0.05, ** *p* < 0.01).

To understand *VyTRXy*’s role in reducing hydrogen peroxide generation, we examined the activity of related oxidoreductases in transgenic plants. After drought stress treatment, the activities of enzymes including ascorbate peroxidase (APX), glutathione peroxidase (GPX), dehydroascorbate reductase (DHAR), and glutathione reductase (GR) were markedly higher in transgenic plants compared to controls (Figure 8). This suggests that *VyTRXy* might decrease H_2_O_2_ production by elevating the activities of APX, GPX, DHAR, and GR. Additionally, we assessed TPX and TrxR levels in transgenic plants. Following 10 days of drought treatment, transgenic plants exhibited a significantly higher TPX content compared to WT plants, while TrxR content was marginally elevated. These findings indicate that *VyTRXy* overexpression augments plants’ antioxidant capacity under drought conditions (Figure 9).

## 3. Discussion

When plants experience drought stress, they generate a toxic substance known as ROS, leading to oxidative damage in cell tissues and disruptions in their physiological and molecular processes [20]. TRXy, a powerful class of peroxide-reducing proteins, is believed to have a critical role in antioxidant systems [21]. In our research, we cloned the *VyTRXy* gene from grapes, and a multiple sequence alignment revealed a highly conserved structural domain typical of the TRX family in VyTRXy (Figure 2A). Subcellular localization studies indicated that VyTRXy primarily resides in chloroplasts (Figure 2D). Specific stress conditions—low temperature, drought, and salt—were observed to induce the expression of MtTrx21, MtTrx27, and MtTrx29 in *Medicago truncatula* [22]. Overexpression of *MsTRX* in tobacco plants resulted in a rise in proline levels and increased POD activity, suggesting that *MsTRX* could boost salt tolerance by maintaining osmotic balance, eliminating ROS, and modulating the transcription of stress-responsive genes [23]. Our analysis of *VyTRXy* expression patterns under different stress treatments showed that abiotic stress could trigger *VyTRXy* expression, with notable responsiveness to drought stress.

TRXy effectively functions as a reducer for an array of peroxide-reducing proteins, vital for redox signaling and oxidative stress responses, and essential in defending against both biotic and abiotic stresses [24]. We generated transgenic tobacco plants by overexpressing the *VyTRXy* gene. These plants, when subjected to drought stress, displayed a phenotype that was subsequently assessed (Figure 3A). WT tobacco showed increased sensitivity to drought stress, whereas transgenic tobacco exhibited a significantly higher survival rate under identical stress parameters (Figure 3C,D), suggesting *VyTRXy*’s potential in enhancing plant drought resistance. The transgenic tobacco registered a substantial reduction in H_2_O_2_ content under drought stress compared to the control (Figure 7A). Examination of osmoregulatory solute content and antioxidant enzyme activity showed that the transgenic tobacco had notably lower electrolyte leakage and MDA levels under drought stress than the control (Figure 4C,D). Furthermore, proline levels in the transgenic tobacco were significantly elevated compared to the control (Figure 4B). MDA is the ultimate decomposition product of membrane lipid peroxidation and indicates the extent of plant damage under adverse conditions. Proline, an intracytoplasmic osmoregulator, is crucial for plant resilience to abiotic stresses. Past research suggests plants can improve their drought resistance by enhancing antioxidant activity. In our study, we measured the activities of antioxidant enzymes CAT, POD, and SOD, discovering that CAT levels were appreciably higher in transgenic plants than in the control (Figure 7D). Moreover, the activities of APX, GPX, DHAR, and GR enzymes were markedly increased in transgenic plants under drought stress (Figure 8). These results imply that VyTRXy may indirectly curtail H_2_O_2_ production by augmenting CAT enzyme activity, thereby engaging the ROS pathway. Additionally, VyTRXy appears to bolster plant drought tolerance by amplifying antioxidant enzyme activity, which in turn diminishes H_2_O_2_ content.

TRXs are pivotal in facilitating redox reactions, regulating photosynthesis and carbohydrate metabolism, responding to abiotic stresses, and preserving a balanced redox state in organisms under stress [25,26,27]. Notably, chlorophyll content significantly diminishes under drought stress [28]. However, in this study, transgenic plants demonstrated considerably higher chlorophyll levels compared to the control group (Figure 4A). They also showed elevated levels of Pn, Gs, Ci, and Tr (Figure 5A–D), suggesting that *VyTRXy* overexpression might boost photosynthesis in plants. FV/FM, indicative of the maximum potential photosynthetic capacity of plant leaves following sufficient dark adaptation, tends to decrease under harsh stress conditions [29]. The transgenic tobacco presented a notably lower F0 content than the WT (Figure 5E), while Fv/Fm and Y(II) were significantly greater in the transgenic tobacco compared to WT (Figure 5E,F). These results imply that *VyTRXy* overexpression in transgenic tobacco may enhance photosynthesis by decreasing F0 or increasing Fv/Fm and Y(II).

Dehydration response element-binding proteins (DREBs) are essential in the plant’s response to abiotic stress, particularly by activating gene transcripts that are responsive to drought [30]. The RD29A promoter, sensitive to drought stress, is mainly activated through the ABA-independent pathway [31]. In tobacco, the *NtRD29A* gene responds to various abiotic stresses [32]. To further understand *VyTRXy*’s role under abiotic stress, our study analyzed the expression patterns of stress-responsive genes (*NtRD29A*, *NtDREB*, *NtERD10C*, *NtLEA5*, *NtCDPK2*, and *NtCOR15*) under drought conditions. It was observed that *VyTRXy* overexpression triggered the expression of drought-associated genes, with *NtRD29A* and *NtDREB* genes showing significantly higher expression levels than others (Figure 6A,B). These observations indicate that expressing grapevine *VyTRXy* genes in transgenic tobacco plants elevated the transcript levels of NtRD29A and NtDREB, thereby potentially improving the plants’ drought tolerance.

## 4. Materials and Methods

### 4.1. Plant Material and Treatment

In this study, Chinese wild grapes (*Vitis yeshanensis* ‘Yanshan’) and tobacco (*Nicotiana benthamiana*) were chosen as experimental materials. Chinese wild grapes were grown naturally in the experimental field of Henan University of Science and Technology, grapevine branches were collected from the field for tissue culture, and cuttings were grown on 1/2 MS medium. After 16 d of succession culture, the healthy and uniformly grown seedlings were selected for stress treatment. Grape seedlings were cultivated in a greenhouse with conditions of 25 °C, 75% relative humidity, and a 14 h light/10 h dark photoperiod. Sterilized tobacco seeds were initially grown on MS medium, followed by transfer to a growth chamber with a stable environment of 25 °C, 70% relative humidity, and a 16 h light/8 h dark photoperiod.

Post-sampling, samples were instantaneously frozen in liquid nitrogen and preserved at −80 °C. Experimental seedlings underwent drought and salt stress treatments in a growth chamber with 20% PEG6000 and 200 mM NaCl for durations of 0, 12, 24, 36, and 48 h, respectively. For low-temperature stress tests, seedlings were exposed to 4 °C for intervals of 0, 2, 6, 12, and 24 h, respectively.

### 4.2. Generation and Identification of Transgenic Plants

The cDNA fragment of VyTRXy was amplified by PCR using gene-specific primers, and the accuracy of the gene was confirmed by sequencing after VyTRXy was cloned into the vector. The transgenic tobacco experiments utilized an Agrobacterium-mediated transformation method [33], in which leaves from 4-week-old tobacco plants were sectioned into approximately 0.5 cm × 0.5 cm pieces, cultured on MS medium for 2–3 days and then inoculated with the Agrobacterium tumefaciens strain GV3101, which carried the recombinant plasmid of VyTRXy. Screening involved a selection medium containing 50 mg/L kanamycin. Resistant shoots that emerged were subsequently rooted in specialized medium before being planted in soil.

Total RNA from transgenic tobacco was isolated using the RNAprep Pure Plant Kit (TIANGEN, Beijing, China), and cDNA was synthesized from the total RNA via the PrimeScriptII1st Strand cDNA Synthesis Kit (TaKaRa, Beijing, China), followed by a PCR assay.

### 4.3. Bioinformatics Analysis of VyTRXy

The protein’s conserved structure was anticipated using the online Motif Scan website (https://myhits.isb-sib.ch/cgi-bin/PFSCAN, accessed on 24 June 2022). The VyTRXy protein’s molecular weight was estimated through the Expasy website (https://web.expasy.org/cgi-bin/protparam/protparam, accessed on 24 June 2022). The VyTRXy protein sequence was retrieved using the NCBI database’s BLASTP tool. A multiple sequence alignment of AtTRXy, OsTRXy, and VyTRXy homologous proteins was executed using the DANMAN 9.0 software (LynnonBiosoft, San Ramon, CA, USA).

### 4.4. Subcellular Localization Analysis

Employing pBI221-GFP as a control plasmid, the fusion plasmid pBI221-GFP/VyTRXy was introduced into Arabidopsis protoplasts via the polyethylene glycol (PEG) transformation method [34]. The transformed samples were then housed in a growth chamber and initially kept in darkness for 18 h. Subsequently, the green fluorescent (GFP) signal was examined under a Zeiss LSM 510 confocal laser scanning microscope (Zeiss, Oberkochen, Germany).

### 4.5. Protein Expression and Enzyme Activity Analysis

The *VyTRXy* gene was inserted into the pGEX-4T-1 vector, positioned between the BamHI and XhoI digestion sites. This construction of the *VyTRXy* prokaryotic expression vector allowed the VyTRXy protein to be expressed in the E. coli BL21 strain. The recombinant proteins were identified using an SDS-PAGE gel kit (Solarbio, Beijing, China), following the manufacturer’s guidelines.

The mutants VyTRXyC96A and VyTRXyC99A were created through PCR-mediated site-directed mutagenesis, substituting cysteine (C) with alanine (A) at positions 96 and 99 within VyTRXy. Primer details for gene cloning are provided in Table S1. Product absorbance at 650 nm was gauged with DDT as a control, and enzyme activity was assessed using a spectrophotometer.

### 4.6. Determination of Photosynthetic Indexes

Measurements of the net photosynthetic rate (Pn), stomatal conductance (Gs), intracellular carbon dioxide concentration (Ci), and transpiration rate (Tr) in fully expanded leaves of transgenic and WT tobacco were taken using a portable photosynthetic system (LI-COR, LI-6400XT, USA). These measurements occurred under clear skies over three consecutive days, from 9:00 am to 4:00 pm, with three consistent data points recorded each time and the average value calculated.

Chlorophyll fluorescence parameters were assessed 2 days post-Pn measurement. Initial (F0) and maximum (Fm) fluorescence yields were captured with leaves in a state of overnight dark adaptation. Variable fluorescence (Fv) was determined as Fv = Fm − F0, and the maximum efficiency of photosystem II photochemistry (PS II) was denoted as Fv/Fm. Non-photochemical quenching (qN) was calculated using the formula qN = 1 − (F’m − F’0)/(Fm − F0), where F’m was assessed in light conditions and F’0 in the dark.

### 4.7. Determination of Physiological Indexes of Transgenic Plants

For the water loss rate, 10 leaves from 3-week-old transgenic and WT plants were immediately weighed upon detachment. These samples were then positioned on dry filter paper in an environment with 40–45% relative humidity at room temperature, then reweighed at intervals of 8, 13, 18, 23, and 28 days. Post-dehydration, the leaves were analyzed for electrolyte leakage, MDA levels, and antioxidant enzyme activities, with pre-dehydration leaves serving as negative controls.

Chlorophyll content was determined by immersing approximately 0.05 g of fresh leaf material in 5 mL of 96% ethanol, followed by an overnight incubation at 4 °C in darkness. Absorbance readings were taken at 665 nm and 649 nm using a spectrophotometer (Hitachi Limited, Tokyo, Japan). The MDA content was determined by selecting mature, uniformly positioned leaves and weighing 0.1 g after punching with a perforator. Avoid the vein area when punching. Lei magnetic DDB-303A portable conductivity meter was used to determine leaf conductivity [35]. Contents of H_2_O_2_, O_2_^−^, proline, MDA, and the activities of SOD, POD, and CAT were evaluated using specific kits (H202-1-Y, A052-1-1, A003-3, A001-4, A007-1, Nanjing, China).

### 4.8. Real-Time Quantitative RT-PCR

Primers for qRT-PCR were devised with Primer Premier 5.0 software. Total RNA was extracted from tobacco and grape leaves using the Spectrum Plant Total RNA Kit (Sigma Aldrich, Beijing, China), and cDNA was synthesized from this RNA using the Prime Script RT Kit (Takara, Dalian, China). The *VvUbi* gene (GenBank sequence number CA808925) was used as the grape’s internal reference gene. The tobacco *NtActin* gene was used as an internal reference. The primers used have been listed in the Supplementary Materials. Experimental results were analyzed using the 2^−ΔΔCt^ method, with each experiment conducted in triplicate.

### 4.9. Statistical Analysis

Data analyses were performed using Excel and SPSS 20.0. One-way analysis of variance with a significance level at α = 0.05 and least significant difference tests were employed to identify significant differences between treatments (*p* < 0.05). All data are presented as mean ± standard error of three biological replicates (*n* = 3).

## 5. Conclusions

This study involved cloning the *VyTRXy* gene from grapes and conducting an enzyme activity assay, confirming its role as a functional TRX. Findings indicate that VyTRXy can enhance photosynthesis, elevate osmoregulator levels, boost antioxidant enzyme activity, and trigger the expression of stress-responsive genes, thereby improving plant drought resistance and reducing damage from environmental stressors. This research provides a basis for further exploration into the functions and mechanisms of the *VyTRXy* gene in grapevines.

## Data Availability

Data are contained within the article and Supplementary Materials.

## Supplementary Materials

The following are available online at https://www.mdpi.com/article/10.3390/ijms242216388/s1.
