# Supplementary material for: Overexpression of Grapevine VyTRXy Improves Drought Tolerance by Maintaining Photosynthesis and Enhancing the Antioxidant and Osmolyte Capacity of Plants
Source: Int J Mol Sci. 2023 Nov 16;24(22):16388. doi: 10.3390/ijms242216388 (PMC10671229; doi:10.3390/ijms242216388)
Supplement: Supplementary file 1 [file ijms-24-16388-s001.zip › ijms-2694996-supplementary.pdf]

Table S1. The sequences of the primers used in these experiments

Primer name	Oligonucleotide primers	Purpose or vector
VyTRXy-F	ATGGCCACCTCGCTCACTTTCTC	PCR
VyTRXy-R	CTATTGTTTCACTTTTAGTGTGG	
VyTRXyq-F	TGATACTGAGAAATACCCGAGCA	qRT-PCR
VyTRXyq-R	CAAAGCACCTTCAAAGCGAT	
VvUbi-F	GTGGTATTATTGAGCCATCCTT	qRT-PCR
VvUbi-R	AACCTCCAATCCAGTCATCTAC	
VyTRXyE-F	GGGGTCGACATGGCCACCTCGCTCACTTTCTC	Expression in
VyTRXyE-R	GGGGGTACCTTGTTTCACTTTTAGTGTGGTCTC	E. coli.
VyTRXy-XbaI-F	GGCTCTAGAATGGCCACCTCGCTCACTTTCTC	pBI221-GFP
VyTRXy-KpnI-R	GCGGGTACCTTGTTTCACTTTTAGTGTGGTCTC	
VyTRXy-NdeI-F	GGGCATATGATGGCCACCTCGCTCACTTTCTC	pGBKT7
VyTRXy-BamHI-R	GGGGGATCCTTGTTTCACTTTTAGTGTGGTCTC	
NtDREB-F	GCCGGAATACACAGGAGAAG	qRT-PCR
NtDREB-R	CCAATTTGGGAACACTGAGG	
NtRD29A-F	TCGGTGTACCAACAGGCATA	qRT-PCR
NtRD29A-R	CCCTTGCTTTGGTGTTGTTT	
NtCOR15A-F	ACGAGCTTAGCAACAAGTT	qRT-PCR
NtCOR15A-R	GGAAATCAAACAAACCACGA	
NtCDPK2-F	AGGTGAGCTTTTCGATAGGATTATT	qRT-PCR
NtCDPK2-R	ACTTCTGGTGCAACATAGTAAGGAC	
NtLEA5-F	TTGTTAGCAGGCGTGGGTAT	qRT-PCR
NtLEA5-R	CTCTCGCTCTTGTTGGGTTC	
NtERD10C-F	AACGTGGAGGCTACAGATCG	qRT-PCR
NtERD10C-R	GTTCCTCTTGGGCATGAGTT	
NtActin-F	CAAGGAAATCACCGCTTTGG	qRT-PCR
NtActin-R	AAGGGATGCGAGGATGGA	

underlined to indicate the digestion site
